# A Non-Invasive Method Based on Computer Vision for Grapevine Cluster Compactness Assessment Using a Mobile Sensing Platform under Field Conditions

**DOI:** 10.3390/s19173799

**Published:** 2019-09-02

**Authors:** Fernando Palacios, Maria P. Diago, Javier Tardaguila

**Affiliations:** 1 Televitis Research Group, University of La Rioja, 26006 Logroño (La Rioja), Spain; 2 Instituto de Ciencias de la Vid y del Vino, University of La Rioja, CSIC, Gobierno de La Rioja, 26007 Logroño, Spain

**Keywords:** image analysis, cluster morphology, RGB, machine learning, non-invasive sensing technologies, proximal sensing, precision viticulture

## Abstract

Grapevine cluster compactness affects grape composition, fungal disease incidence, and wine quality. Thus far, cluster compactness assessment has been based on visual inspection performed by trained evaluators with very scarce application in the wine industry. The goal of this work was to develop a new, non-invasive method based on the combination of computer vision and machine learning technology for cluster compactness assessment under field conditions from on-the-go red, green, blue (RGB) image acquisition. A mobile sensing platform was used to automatically capture RGB images of grapevine canopies and fruiting zones at night using artificial illumination. Likewise, a set of 195 clusters of four red grapevine varieties of three commercial vineyards were photographed during several years one week prior to harvest. After image acquisition, cluster compactness was evaluated by a group of 15 experts in the laboratory following the International Organization of Vine and Wine (OIV) 204 standard as a reference method. The developed algorithm comprises several steps, including an initial, semi-supervised image segmentation, followed by automated cluster detection and automated compactness estimation using a Gaussian process regression model. Calibration (95 clusters were used as a training set and 100 clusters as the test set) and leave-one-out cross-validation models (LOOCV; performed on the whole 195 clusters set) were elaborated. For these, determination coefficient (R^2^) of 0.68 and a root mean squared error (RMSE) of 0.96 were obtained on the test set between the image-based compactness estimated values and the average of the evaluators’ ratings (in the range from 1–9). Additionally, the leave-one-out cross-validation yielded a R^2^ of 0.70 and an RMSE of 1.11. The results show that the newly developed computer vision based method could be commercially applied by the wine industry for efficient cluster compactness estimation from RGB on-the-go image acquisition platforms in commercial vineyards.

## 1. Introduction

Grapevine cluster compactness is a key attribute related to grape composition, fruit health status, and wine quality [1,2]. Compactness defines the density of the cluster by the degree of the aggregation of its berries. Highly compacted winegrape clusters can be affected to a greater extent by fungal diseases, such as powdery mildew [3] and botrytis [4], than loose ones [5].

The most prevalent method for assessing cluster compactness was developed by the International Organization of Vine and Wine (OIV) [6] and has been applied in several research studies [7,8]. This OIV method procures cluster compactness assessment by visual inspection in five different classes. This compactness class takes into account several morphological features of the berries and pedicels, which are visually appraised by trained experts. This method and others designed to evaluate compactness on specific varieties [9,10,11] tend to be inaccurate due to the intrinsic subjectivity of the evaluation linked to the evaluator’s opinion. Moreover, these visual inspection methods are laborious and time-consuming, as they may also require the manual measurement of specific cluster morphological parameters. Therefore, alternative methods for objectively and accurately assessing cluster compactness are needed for wine industry applications.

Computer vision and image processing technology enables low-cost, automated information extraction and its analysis from images taken using a digital camera. This technology is being used in viticulture to estimate key parameters such as vine pruning weight [12,13], the number of flowers per inflorescence [14,15], canopy features [16], or yield [17,18], as well as to provide relevant information to grape harvesting robots [19,20].

Automated cluster compactness estimation by computer vision methods was recently attempted by Cubero et al. [21] and Chen et al. [22]. The former involved the automated extraction of image descriptors from red, green, blue (RGB) cluster images taken from different cluster views under laboratory conditions. From these descriptors, a partial least squares (PLS) calibration model was developed to predict their associated OIV compactness rating. In the approach followed by Chen et al. [22], a multi-perspective imaging system was developed, which made use of different mirror reflections that facilitated the simultaneous acquisition of images from multiple views from a single shot. Additionally, the system also included a weighing sensor for cluster mass measurement. Then, a set of image descriptors and features derived from the data provided by the sensing system were automatically extracted and used to calibrate several models. Of these, the PLS model achieved the best results.

Previously developed computer vision methods for cluster compactness assessment provided accurate and objective compactness estimation only working under controlled laboratory conditions, which requires the destructive collection of clusters in the vineyard. This is a laborious and time-consuming practice that precludes the appraisal of cluster compactness as a standard grape quality parameter prior to harvest, thus limiting its industrial applicability. Moreover, to the best of our knowledge, there is no commercial method available to assess grapevine cluster compactness under field conditions in an automated way.

The purpose of this work was to develop a new, non-invasive, and proximal method based on computer vision and machine learning technology for assessing grapevine cluster compactness from on-the-go RGB image acquisition in commercial vineyards.

## 2. Materials and Methods

### 2.1. Experimental Layout

The trials were carried out during seasons 2016, 2017, and 2018 in three commercial vineyards planted with four different red grapevine varieties (*Vitis vinifera* L). The vines were trained onto a vertical shoot positioned (VSP) trellis system and were partially defoliated at fruit set.

An overall set of 195 red grape clusters involving five distinct datasets were labeled in the field prior to image acquisition in three commercial vineyards.

- Vineyard site #1: Located in Logroño (lat. 42°27′42.3″N; long. 2°25′40.4″W; La Rioja, Spain) with 2.8 m row spacing and 1.2 m vine spacing, where a set of 95 Tempranillo clusters were imaged and sampled during season 2016, denoted as T16.
- Vineyard site #2: Located in Logroño (lat. 42°28′34.2″N; long. 2°29′10.0″W; La Rioja, Spain) with 2.5 m row spacing and 1 m vine spacing, where a set of 25 Grenache clusters were imaged and sampled during season 2017, denoted as G17.
- Vineyard site #3: Located in Vergalijo (lat. 42°27’46.0” N; long. 1°48’13.1” W; Navarra, Spain) with 2 m row spacing and 1 m vine spacing, where three sets of 75 clusters of Syrah, Cabernet Sauvignon, and Tempranillo (25 per grapevine variety) were imaged and sampled during season 2018, denoted as S18, CS18, and T18, respectively.

The data were divided into a training set, formed by 95 clusters of T16 dataset, and an external validation test set, formed by 100 clusters of G17, S18, CS18, and T18 datasets, in order to test the system performance on new or additional varieties and vineyards.

### 2.2. Image Acquisition

**Figure 1:**
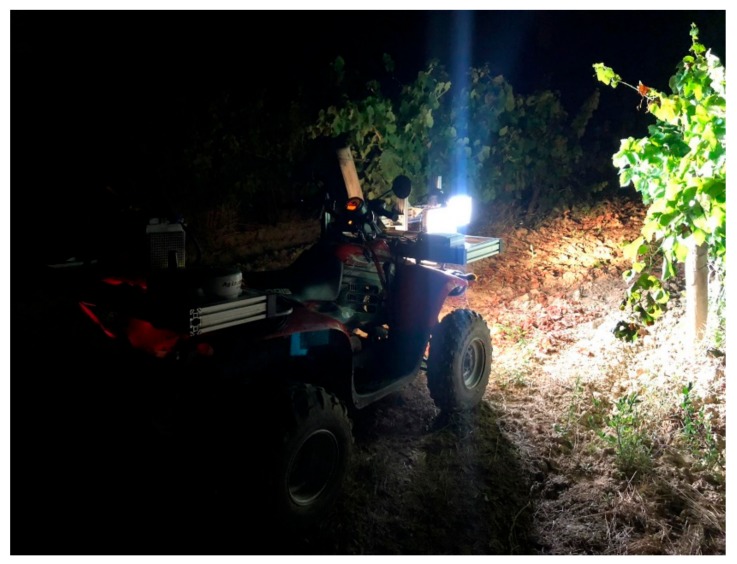
Mobile sensing platform for on-the-go image acquisition: a modified all-terrain vehicle (ATV) incorporating a red, green, blue (RGB) camera, Global Positioning System (GPS), and an artificial illumination system mounted on an adaptable structure.

Vineyard canopy images were taken on-the-go at a speed of 5 Km/h one week prior to harvest using a mobile sensing platform developed at the University of La Rioja. Image acquisition was performed at night using an artificial illumination system mounted onto the mobile platform in order to obtain homogeneity on the illumination of the vines and to separate the vine under evaluation from the vines of the opposite row. An all-terrain vehicle (ATV) (Trail Boss 330, Polaris Industries, Medina, Minnesota, USA) was modified to incorporate all components as described in the work of Diago et al. [23] (Figure 1). 

Additionally, some elements were modified:

- RGB camera: a mirrorless Sony α7II RGB camera (Sony Corp., Tokyo, Japan) mounting a full-frame complementary metal oxide semiconductor (CMOS) sensor (35 mm and 24.3 MP resolution) and equipped with a Zeiss 24/70 mm lens was used for image acquisition in vineyard sites #1 and #2, while a Canon EOS 5D Mark IV RGB camera (Canon Inc. Tokyo, Japan) mounting a full-frame CMOS sensor (35 mm and 30.4 MP) equipped with a Canon EF 35 mm F/2 IS USM lens was used in vineyard site #3.
- Industrial computer: A Nuvo-3100VTC industrial computer was used for image storage and camera parameters setting for the Canon EOS 5D Mark IV using custom software developed, while the parameters of the Sony α7II camera were set in the camera itself and the storage in a Secure Digital (SD)-card.

The camera was positioned at a distance of 1.5 m from the canopy. The camera parameters were manually set at the beginning of the experiment in each vineyard.

### 2.3. Reference Measurements of Cluster Compactness

**Figure 2:**
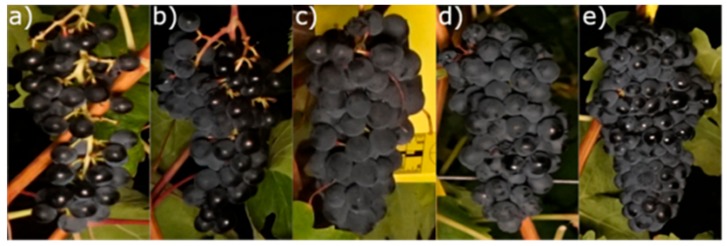
Examples of clusters with different compactness ratings according to the International Organization of Vine and Wine (OIV) 204 standard: class 1 (**a**) very loose clusters; class 3 (**b**) loose clusters; class 5 (**c**) medium compact clusters; class 7 (**d**); compact clusters; class 9 (**e**) very compact clusters.

After image acquisition, the labeled clusters were manually collected, and their compactness was visually evaluated in a laboratory at the University of La Rioja by a panel composed of 15 experts following the OIV 204 standard [6]. In this reference method, each cluster was classified in one of five discrete classes (Figure 2) ranging from 1, the loosest clusters, to 9, the most compact clusters. In the visual assessment, several aspects related to the morphology of the cluster, such as berries’ mobility, pedicels’ visibility, and berries’ deformation by pressure, were taken into consideration. The average of the evaluators’ ratings was used as the reference compactness value for each cluster.

### 2.4. Image Processing

**Figure 3:**
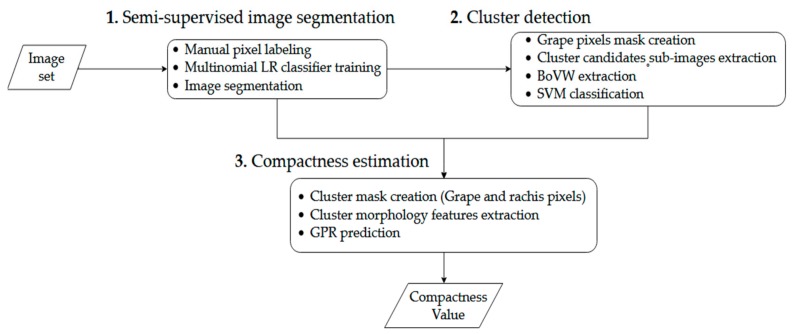
Flow-chart of the full algorithm for a new set of images. First, a set of pixel labeling was required to train a multinomial logistic regression model to segment the whole set. Second, the cluster candidates were extracted and filtered using a bag of visual words model. Finally, compactness features were extracted, and the estimation was performed on each cluster by the Gaussian process regression model.

Image processing comprised several steps that can be summarized as semi-supervised image segmentation followed by cluster detection and compactness estimation for each detected cluster. While cluster detection and compactness estimation were fully automated, the image segmentation step required the intervention of the user for each dataset. The algorithm was developed and tested using Matlab R2017b (Mathworks, Natick, MA, USA). The flowchart of the algorithm process for a new set of images is described in Figure 3.

To ensure consistency in the analysis of the complete algorithm, the classifier used at each step was trained using the training set and evaluated with the output obtained by the classifier of the previous step for the test set, except for the initial image segmentation, where a model was trained on each individual dataset.

#### 2.4.1. Semi-Supervised Image Segmentation

For the proper compactness estimation of every cluster visible in the image, a previous detection of the clusters and their main elements (grape and rachis) was needed.

Most of the red winegrape pixels are easily distinguishable from pixels of other vine elements by their color. Hence, an initial pixelwise color-based segmentation was performed on every image. For this approach, seven classes representing the elements of the grapevines potentially present in their images were defined: “grape”, “rachis”, “trunk”, “shoot”, “leaf”, “gap”, and “trellis”. For extracting cluster candidates, only groups of pixels belonging to the first class (“grape”) were used, but rachis identification was also relevant for compactness assessment. In summary, an image segmentation considering the seven classes described above as a first step eliminated the necessity of further color segmentation.

A set of 3500 pixels were manually labeled (500 pixels per class), and color features were extracted considering a combination of two color spaces: RGB and CIE L*a*b* (CIELAB) [24]. In this approach, a pixel p was mathematically represented as in Equation (1):

(1) $$p=\left[R_{p},G_{p},B_{p},L_{p},a_{p},b_{p}\right]$$

where $R_{p},G_{p},B_{p}$ and $L_{p},a_{p},b_{p}$ represent the values of p for the three channels of the *RGB* and the *CIELAB* color spaces, respectively. The function rgb2lab from Matlab R2017b was used for the RGB to CIELAB color space conversion.

A multinomial logistic regression was trained with the set described above in order to obtain a pixel wise color based classifier. This classifier, which is a generalization of logistic regression for multiclass problems [25], predicts the probability of each possible outcome for an observation as the relative probability of belonging to each class over belonging to another one chosen arbitrarily as the reference class. Assuming that n is the reference class of a set of $\left\{1,\ldots ,\;n\right\}$ classes, the output of the classifier for p is $\left[\pi_{1},\ldots ,\pi_{n}\right]$, where $\pi_{i}$ represents the probability of p belonging to the class i for i=1,…,n−1 [Equation (2)], and $\pi_{n}$ represents the probability for the reference class [Equation (3)].

(2) $$\pi_{i}=\frac{e^{\beta_{i,0}+\sum_{j=1}^{k}\beta_{i,j}x_{j}}}{1+\sum_{l=1}^{n-1}e^{\beta_{l,0}+\sum_{j=1}^{k}\beta_{l,j}x_{j}}}$$

(3) $$\pi_{n}=\frac{1}{1+\sum_{l=1}^{n-1}e^{\beta_{l,0}+\sum_{j=1}^{k}\beta_{l,j}x_{j}}}=1-\overset{n-1}{\underset{l=1}{\sum }}\pi_{l}$$

where k is the number of predictor variables, $x_{j}$ the j-th predictor variable, and $\beta_{i,j}$ is the estimated coefficient for the *i*-th class.

**Figure 4:**
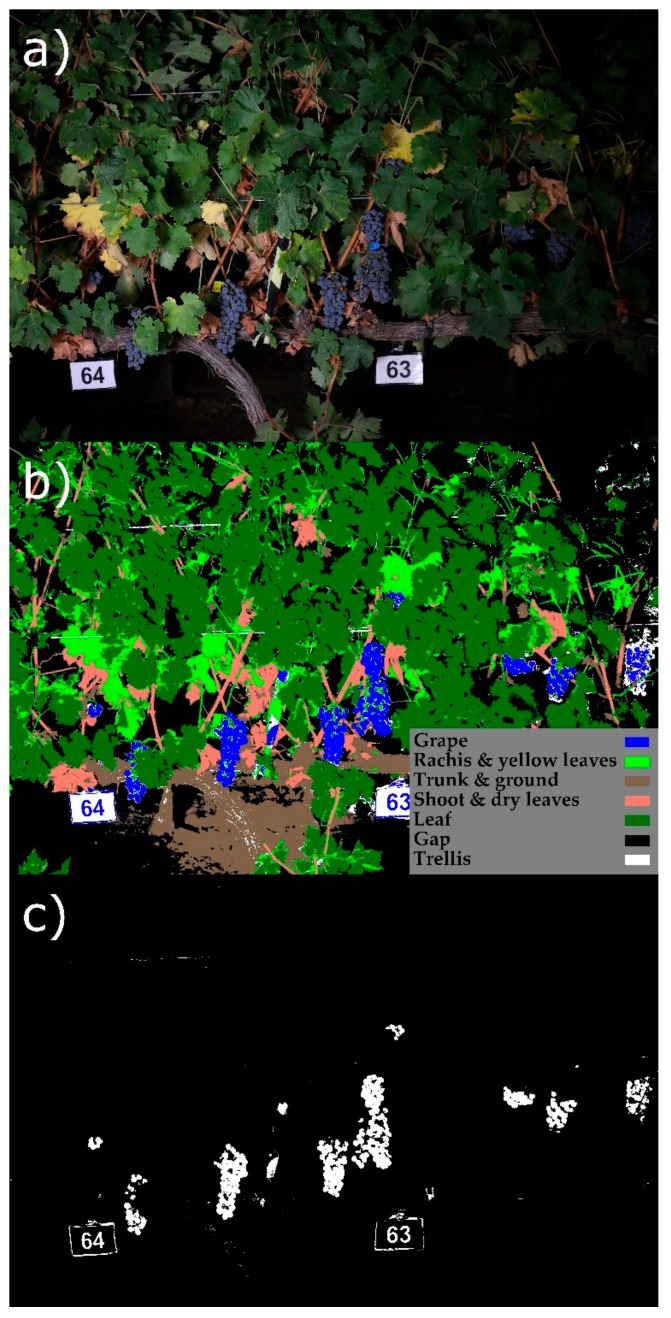
Initial semi supervised segmentation. A set of pixels were manually labeled on the set of the original images (**a**) into seven predefined classes (“grape”, “rachis”, ‘trunk”, “shoot”, “leaf”, “gap”, or “trellis”), and the multinomial logistic regression model performed the segmentation on the whole set of images. (**b**) Some pixels of elements without a predefined class were misclassified (e.g., yellow leaves identified as “rachis”, dry leaves identified as “shoot”, or ground identified as “trunk”). The pixels classified as “grape” and marked in white (**c**) were used for identifying cluster candidates.

The segmentation was performed by assigning to each pixel the class with the highest probability (Figure 4).

#### 2.4.2. Cluster Detection

Using the initial segmentation, a mask of cluster candidates was then generated by selecting those pixels assigned to the “grape” class (Figure 4c).

While the initial color segmentation allowed filtering most of the non-cluster elements presented in the image, a second filtering step was needed to remove those non-cluster groups of pixels with similar color to the “grape” class, which can form objects with different shapes and sizes. This second filtering can be summarized as:

- A morphological opening (morphological erosion operation followed by a dilation) of the clusters’ candidates mask using a circular kernel with a radius of three pixels.
- An extraction of a sub-image per minimal bounding box that contains a connected component (groups of connected pixels) in the clusters’ candidates mask.
- An extraction of features for each sub-image that represents the information contained on it. For this, the bag-of-visual-words (BoVW) was employed.
- A classification of “cluster” vs. “non-cluster” sub-images.

The bag-of-visual-words (BoVW) model is a concept derived from document classification for image classification and object categorization [26]. In this model, images are treated as documents formed by local features denominated “visual words”. These words are grouped to form a “vocabulary” or “codebook”. Then, every image is represented by the number of occurrences of every “codeword” in the codebook. In this work, local features were 64-length speeded up robust features (SURF) descriptor vectors [27] clustered by a *k*-means algorithm [28].

Given a set of *n* training sub-images, *Tr*, represented as $Tr=\left\{tr_{1},\ldots ,tr_{n}\right\}$ and their class $Y=\left\{y_{1},\ldots ,y_{n}\right\}$ manually labeled into “cluster” (total and partial clusters) and “non-cluster”, the process adopted for the training set is the following:

1. To extract SURF points for every sub-image.
2. Cluster SURF points applying *k*-means. The set of cluster centroids would form the codebook of *k* codewords.
3. Extraction of the bag-of-words per sub-image:
  - To assign each SURF point of the image to the nearest centroid of the codebook.
  - To calculate the histogram by counting the number of SURF points assigned to each centroid.

**Figure 5:**
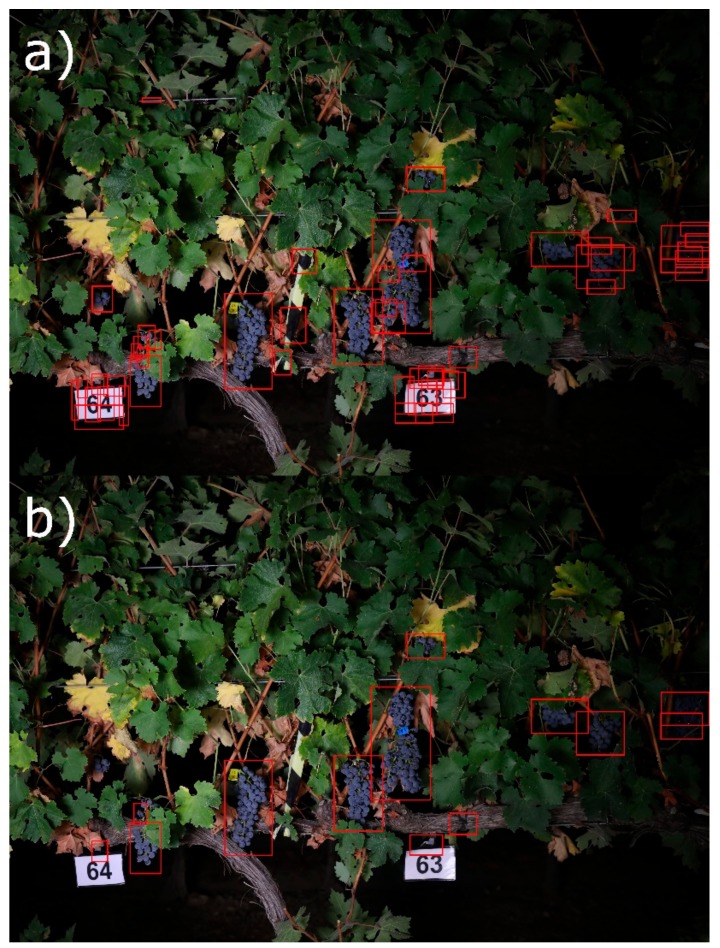
Cluster candidates’ extraction and filtering. (**a**) Bounding boxes were extracted from connected components of grape pixels and (**b**) filtered using the bag-of-visual-words (BOVW) model to estimate the cluster compactness of the final non-filtered regions.

Then, *tr_i_* had a feature vector $x_{i}\in \;{\mathbb{R}}^{k}$ that was used to train a support vector machine (SVM) classifier [29]. This is a machine learning algorithm for supervised learning classification or regression tasks that transforms input data into a high-dimensional feature space using a kernel function and finds the hyperplane that maximizes the distance to the nearest training data point of any class. With the classifier trained, only step 3 was performed for new sets of cluster candidates sub-images, and the resulting feature vectors were used to classify each sub-image into “cluster” or “non-cluster” classes, preserving only cluster sub-images for further analysis (Figure 5).

#### 2.4.3. Cluster Compactness Estimation

This step involved the extraction of a set of features from the cluster morphology that were related to its compactness. For that purpose, the segmented pixels corresponding to the initial segmentation mask of the sub-images classified as clusters in the previous step had to be extracted. For a given detected cluster sub-image, the next procedure was followed:

- A new mask using only pixels of “grape” and “rachis” classes was created.
- A morphological opening on “grape” pixels using a circular kernel with a radius of two pixels was applied.
- A morphological opening on “rachis” pixels using a circular kernel with a radius of two pixels was also applied.
- A mask containing only the largest connected component formed by “grape” and “rachis” pixels, denoted as mask “A”, was created.
- A mask containing the convex hulls of each “grape” pixel’s connected component (that can represent several grouped berries on compact clusters or isolated grapes on loose clusters), denoted as mask “B”, was created.
- The final mask was created containing “grape” pixels and “rachis” pixels that were in mask “A” and inside the region of the convex hull of mask “B”. Those “rachis” pixels in mask “A” that were outside of the convex hull of mask “B” and connected at least two connected components of “grape” pixels were included as well.

**Figure 6:**
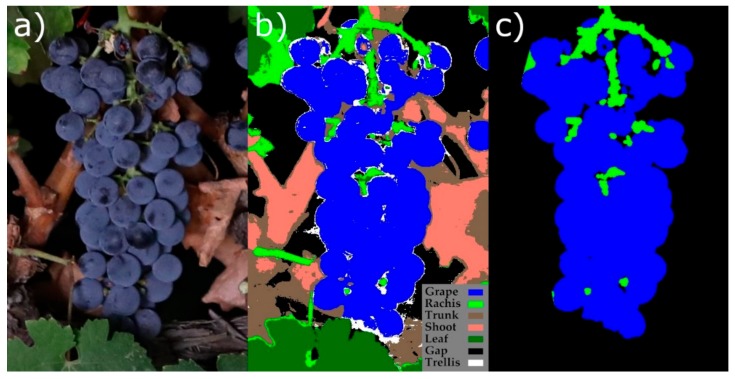
Extraction of the clusters’ final masks for compactness estimation. (**a**) Extracted cluster sub-image, (**b**) its corresponding segmentation, and (**c**) a cluster mask obtained using “grape” and “rachis” pixels and morphological operations.

The features to estimate the cluster compactness were extracted from the last mask containing only “grape” and “rachis” pixels (Figure 6). These features were the following:

- Ratio of the area of the convex hull body of the cluster corresponding to holes (AH)
- Ratio of the clusters area corresponding to berries (AB)
- Ratio of the area corresponding to “rachis” (AR)
- Average width at 25±5% of the length of the cluster (W25)
- Average width at 50±5% of the length of the cluster (W50)
- Average width at 75±5% of the length of the cluster (W75)
- Ratio between “rachis” and “grape” pixels (RatioRG)
- Roundness of “grape” pixels (RDGrape): $\frac{4.0\;\times \;\pi \;\times \;A_{Grape}}{P_{Grape}{}^{2}}$
- Compactness shape factor of “grape” pixels (CSFGrape): $\frac{P_{Grape}{}^{2}}{A_{Grape}}$
- Ratio between the maximum width and the length of the cluster (AS)
- Ratio between *W75* and *W25* (RatioW75_W25)
- Proportion of the “rachis” pixels “inside” the cluster (*RR_in_*)
- Proportion of the “rachis” pixels “outside” the cluster (*RR_out_*)
- Ratio of the area of the cluster over the mean area of the clusters of its set (*R_AoM_*)

Where $A_{Grape}$ and $P_{Grape}$ correspond to the area and the perimeter, considering only grape pixels.

While some of these features were already addressed by Cubero et al. [21] and Chen et al. [22], they had to be adapted to the new environmental situation of field conditions, while others were specifically designed for this study. Features based on clusters’ widths and lengths (*W25*, *W50*, *W75*) required a prior rotation of the cluster mask along the longest axis to match the width of the cluster with the horizontal axis, thus the whole set of clusters could have a similar orientation. The features *RR_in_* and *RR_out_* were calculated as the proportion of “rachis” pixels completely surrounded by “grape” pixels, and the rest of the “rachis” pixels that were not, respectively. The feature *R_AoM_* provided a measure about the size of the cluster over the average of the clusters on its set and added robustness by incorporating images of clusters taken at different distances.

The compactness estimation was performed by a Gaussian process regression (GPR) model trained with the data extracted from *n* clusters ${\left\{\left(x_{i},y_{i}\right)\right\}}_{i=1}^{n}$ where $x_{i}\in {\mathbb{R}}^{14}$ represents the 14-feature vector, and $y_{i}\in \left[1,9\right]$ represents the average of the ratings of the evaluators for the *i*-th cluster.

Gaussian process regression models are probabilistic kernel-based machine learning models that use a Bayesian approach to solve regression problems estimating uncertainty at predictions [30]. A Gaussian process regression model is described in Equation (4):

(4) $$g\left(x\right)=f\left(x\right)+h{\left(x\right)}^{T}\beta$$

where $h\left(x\right)$ is a vector of basis functions, β is the coefficient of $h\left(x\right)$, and $\;f\left(x\right)\;\sim \;GP\left(0,k\left(x,x^{\prime }\right)\right)$ is a zero mean Gaussian process with a $k\left(x,x^{\prime }\right)$ covariate function.

#### 2.4.4. Performance Evaluation Metrics

The results obtained for each step were analyzed using a set of metrics corresponding to classification tasks in the case of multinomial logistic regression and support vector machine and regression for the Gaussian process regression. The metrics chosen for classification performance are commonly used for evaluating results of binary classifiers, where a sample can be identified as positive class or negative class. For multinomial logistic regression, the positive and the negative classes were the class under evaluation and the rest of them (e.g., “grape” class vs. “non-grape” classes), while for support vector machine, the “cluster” and the “non-cluster” classes were considered, respectively. The metrics calculated were sensitivity [Equation (5)], specificity [Equation (6)], F1 score [Equation (7)], and intersect over union [IoU; Equation (8)].

(5) $$Sensitivity=\frac{TP\;}{TP+FN}$$

(6) $$Specificity=\frac{TN\;}{TN+FP}$$

(7) $$F_{1}=2\times \;\frac{Precision\times Sensitivity\;}{Precision+Sensitivity}$$

(8) $$IoU=\frac{TP\;}{TP+FP+FN}$$

where *TP* represents the “true positives” (number of positive samples correctly classified as positive class), *FP* represents the “false positives” (number of negative samples incorrectly classified as positive class), *TN* represents the “true negatives” (number of negative samples correctly classified as negative class), and *FN* represents the “false negatives” (number of positive samples incorrectly classified as negative class). *Precision* was defined as in Equation (9):

(9) $$Precision=\frac{TP\;}{TP\;+\;FP}$$

The area under the receiver operating characteristic (ROC) curve (AUC) [31] was also considered. For regression, the determination coefficient (R^2^) and the root mean squared error (RMSE) were selected.

#### 2.4.5. Hyperparameter’s Optimization Procedure

Support vector machine and Gaussian process regression are two machine learning algorithms that have a set of hyperparameters that are not learned from the data and need to be set before the training. The most traditional hyperparameter selection method is a brute-force grid search of the best subset of hyperparameters combined with a manually predefined set of values established for each hyperparameter in order to optimize a performance metric. Instead, in this work, a Bayesian optimization algorithm [32] was used for finding the best hyperparameters set, which proved to outperform other optimization algorithms [33]. This algorithm finds the best hyperparameter set that optimizes an objective function (in this context, a performance metric of the machine learning algorithm) using a Gaussian process trained with the objective function evaluations. The Gaussian process is updated with the result of each evaluation of the objective function, and an acquisition function is used to determine the next point to be evaluated in a bounded domain, i.e., the next set of hyperparameters.

The functions fitcsvm and fitrgp of Matlab R2017b were used to train the support vector machine and the Gaussian process regression models, respectively, selecting their hyperparameters with Bayesian optimization. A Gaussian process with automatic relevance determination (ARD) Matérn 5/2 kernel model and the expected-improvement-plus acquisition function were used for this purpose. The ranges considered for each hyperparameter and the final values are shown in Table 1.

## 3. Results and Discussion

### 3.1. Initial Segmentation Performance

The initial segmentation process was a key step towards the accurate assessment of cluster compactness. Therefore, a different segmentation model was applied to each grapevine variety and vineyard to avoid errors associated with slight differences in color and illumination from the images captured from one vineyard to another, which would occur if applying a unique segmentation model. Likewise, five sets of 3500 pixels each were manually labeled (500 pixels per class) for each variety and vineyard, which were used to train five distinct multinomial logistic regression models.

As shown in Table 2, overall, the five models achieved good results in terms of sensitivity, specificity, F1 score, AUC, and IoU when applied to their specific set of images. With regard to the most relevant classes (“rachis” and “grape”), similar and equally good values were obtained for specificity for all sets, while more variable outcomes were obtained for the remaining metrics. In general terms, the T18 model yielded the best results for these two relevant classes, closely followed by the S18 model, in this case only for the “grape” class, and slightly outperformed by the CS18 model for the “rachis” class in terms of sensitivity and AUC. More modest results were obtained for models G17 and T16 (Table 1). Particularly, model T16 yielded values under 0.9 in sensitivity and F1 score metrics for the “rachis” class and in IoU for both “rachis” and “grape” classes.

To compare the segmentation performance between individual models on each dataset versus a unique segmentation model, two additional cross-validation methods were applied: a five-fold cross-validation, where at each iteration, the training fold was formed by four datasets and the test fold by the remaining dataset, and a ten-fold stratified cross-validation, where at each iteration, the training and the test folds comprised data at an equal proportion of each dataset and class. The comparison of the results for these two validation methods revealed that better results were obtained for all metrics when the training and the test contained data from the same dataset (ten-fold CV). Comparing both methods with the average of the results obtained by the individual models indicated that applying individual segmentation models produced a substantial improvement in all metrics (except for specificity, for which only a slight increase was recorded) and for all classes (with the exception of the “gap” class, for which similar results were obtained using the three methods). The increase in these metrics for the relevant classes (“grape” and “rachis”) and the importance of this step highlights the need for applying individual models for each dataset. Differences between performances could be related to differences in color tonality of the vine elements segmented (e.g., different green tonalities for leaves) between grape varieties and vineyards.

The results show that, given a set of images taken on a vineyard, a multinomial logistic regression model trained with a small subset of pixels manually labeled from the images can be applied to effectively segment vine images in the predefined classes using color information. Also, the pixels needed for compactness estimation (“grape” and “rachis” pixels) can be extracted.

### 3.2. Cluster Detection Performance

A support vector machine was trained with 600 sub-images manually labeled into 300 “cluster” (total and partial clusters) and 300 “non-cluster” sub-images automatically extracted from the segmentation performed on the T16 set.

The classifier was validated against a set of 800 sub-images automatically extracted from the segmentation performed on sets G17, S18, CS18, and T18 (200 sub-images per set) and manually labeled into 400 “cluster” (total and partial clusters) and 400 “non-cluster” sub-images (100 sub-images of each class per set). A set of *k* values in a range from 10–200 was chosen for the *k*-means algorithm, and the performance of the classifier for the “cluster” class was evaluated (Table 3). The model trained with *k* = 100 yielded the best results for all metrics. Similar results were obtained for all *k* values in terms of sensitivity and F1 score, while for specificity and AUC, the model trained with *k* = 10 performed poorer than the rest of models. The model that yielded the best results (*k* = 100) showed similar values in sensitivity, specificity, and F1 (between 0.76 and 0.8), with specificity being slightly superior, while a higher value was obtained for AUC.

These results could be improved by incorporating new data, as is visualized in Table 3. A five-fold cross validation (each fold being a different set) was performed to train new support vector machine classifiers. The results show an improvement over all metrics for all *k* tested values, with the exception of specificity for the *k* = 100 model, whose value was slightly diminished. This model still achieved the best results for all metrics except specificity. The *k* = 100 model was chosen as the final model, as it yielded the best results with the test set in all metrics as well as the best results in almost all metrics at the cross validation.

The performance of the support vector machine proves that this classifier trained with the bag-of-visual-words representation of “cluster” and “non-cluster” sub-images can be applied to classify new sub-images from new datasets previously unknown to the classifier, and therefore, it can be used to filter non-cluster pixel groups before compactness estimation.

### 3.3. Cluster Compactness Estimation

**Figure 7:**
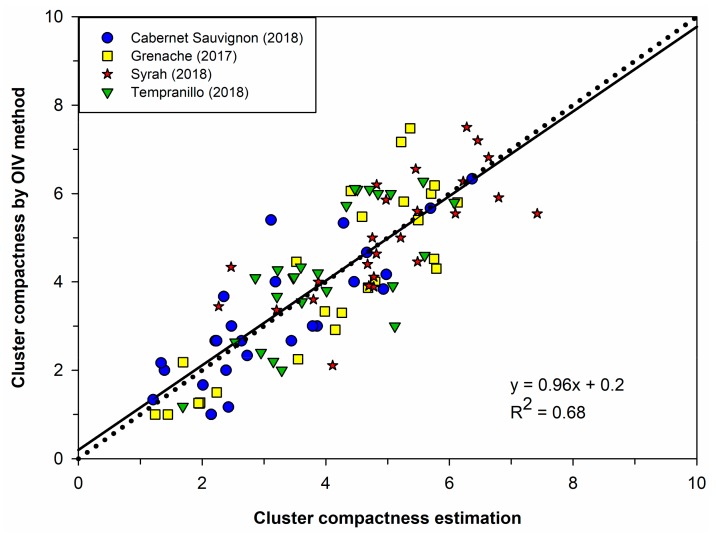
Performance of the GPR model on the test set (100 clusters); correlation between the cluster compactness estimation performed by the model and the OIV ratings (reference method) evaluated visually by the panel of experts.

A Gaussian process regression model was trained on the set of features extracted from the clusters of the T16 set (95 clusters) and validated with the automatically detected clusters of G17, S18, CS18, and T18 sets (100 clusters). A coefficient of determination (R^2^) of 0.68 and an RMSE of 0.96 were achieved (Figure 7). This is a remarkable result, considering that the four test sets were totally unknown to the classifier and were taken on different vineyards. Also, S18, CS18, and T18 test sets were photographed with a different RGB camera than the one used to photograph the T16 training set. Even more relevant is that three of the four sets (75 clusters of 100) were formed by varieties different from the one used for training (Tempranillo was used for training, while Grenache, Syrah, Cabernet Sauvignon, and Tempranillo clusters were included in the test set). This outcome paves a way to cluster compactness estimation of winegrape varieties without the requirement of representing variety in the training data—in contrast to the work presented by Cubero et al. [21]—and paves a way to real context application on new varieties and vineyards without the necessity of including specific data from those varieties and vineyards, which would require retrieving new clusters and assessing their compactness by trained experts.

**Figure 8:**
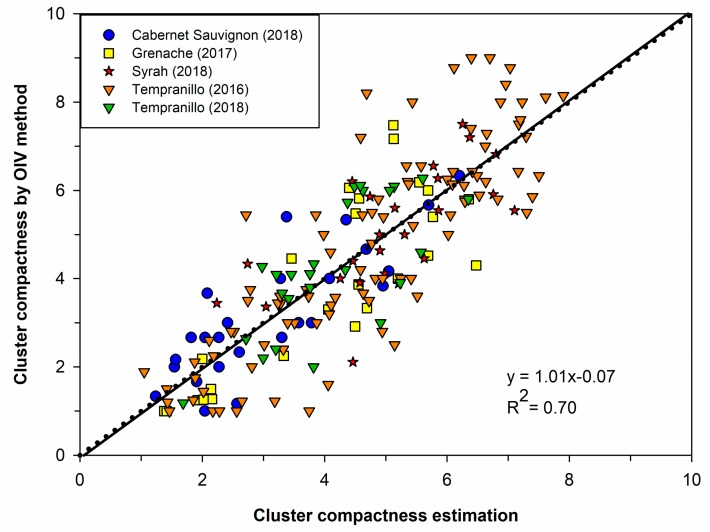
Performance of the GPR model performing leave-one-out cross validation (LOOCV) on the whole data (195 clusters); correlation between the cluster compactness estimation performed by the model and the OIV ratings (reference method) evaluated visually by the panel of experts.

On the other hand, when leave-one-out cross validation over all datasets (195 clusters) was performed, a coefficient of determination (R^2^) of 0.70 and an RMSE of 1.11 were obtained (Figure 8). It can be observed that the algorithm had an accurate performance along most of the compactness range but tended to slightly underestimate highly compacted clusters with an OIV rating close to 9. A feasible reason for this could be that, since this very high compact class was mainly characterized by the deformation of the berries due to the pressure among berries, this feature was difficult to extract by image analysis.

The accuracy of compactness estimation is also meant to be highly influenced by the results obtained in previous steps, i.e., a high misclassification rate in the initial segmentation of cluster and “rachis” pixels would produce wrong shapes in the final cluster mask and a poor feature extraction for the cluster. Also, for estimating the compactness of a given cluster, this has to be previously detected by the BOVW model.

**Figure 9:**
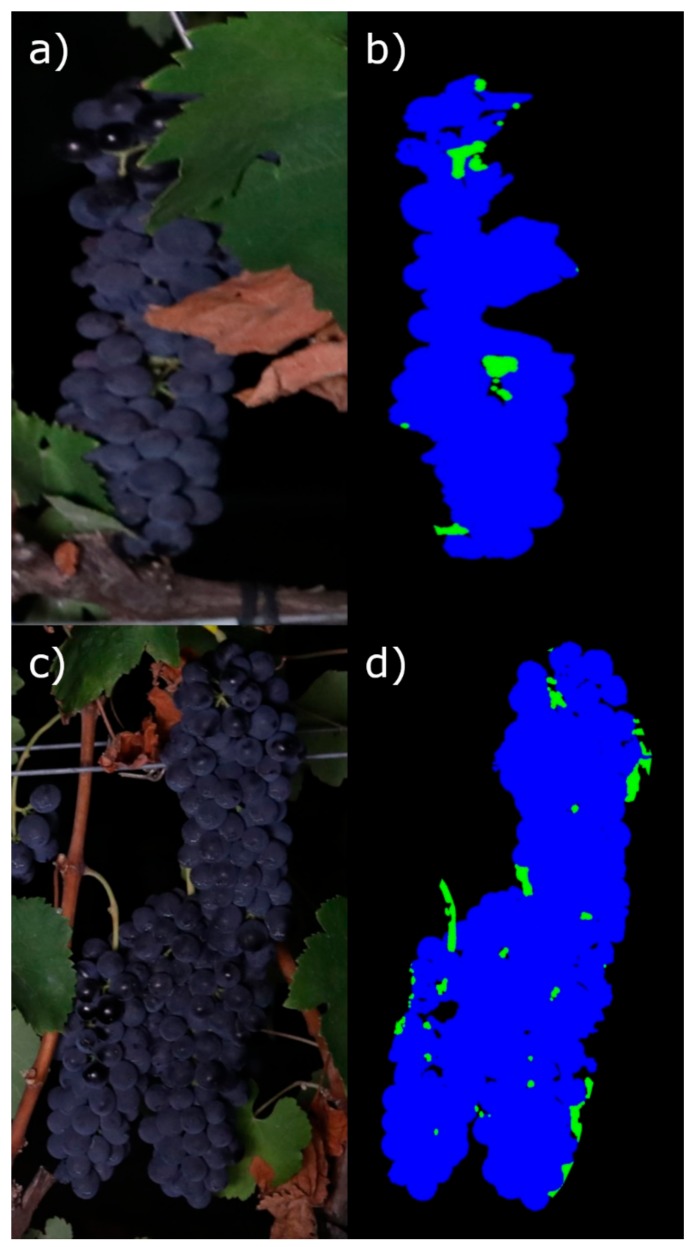
Examples of cluster occlusions and overlapping in commercial vineyards limiting compactness estimation. Cluster partially occluded by leaves (**a**), multiple overlapped clusters (**c**), and final segmented masks used for compactness estimation (**b**,**d**).

The cluster compactness estimation using the developed methodology in this work could be limited by some experimental in-field conditions, as follows:

- Occlusion of the cluster: the estimation was only performed on the visible region of the cluster. Therefore, a high level of occlusion of the cluster could increase the estimation error. An example of a cluster partially occluded by leaves is shown in Figure 9a and the final mask extracted for compactness estimation in Figure 9b, where the cluster mask presents an anomalous shape that would lead to incorrect compactness estimation.
- Cluster overlapping: highly overlapped clusters would be identified as one, and therefore a unique estimation would be obtained, associated with the set of overlapped clusters. An example is illustrated in Figure 9c, where a set of clusters are overlapped, and in the extracted mask (Figure 9d), the clusters cannot be separated from each other for proper individual compactness estimation.

At the current state of the system, the occlusion problem could be overcome by defoliating the side of the vineyard to be photographed. For cluster overlapping, those groups of overlapped clusters could be isolated in the field, or the separation between them could be manually labeled on the images with a clearly different color than “rachis” and “grape” colors (e.g., “trunk” color).

### 3.4. Commercial Applicability

The developed system can be efficiently used to estimate the cluster compactness in commercial vineyards. The image acquisition carried out by a mobile sensing platform allows the user to take a high number of images in extensive vineyards, which can be automatically geo-referenced. Therefore, the geo-referenced compactness estimations could be used to generate a map that illustrates the spatial variability in cluster compactness, which could be very useful to delineate zones according to cluster compactness and to identify those with similar values. This information could be highly relevant for sorting grapes before harvest, as cluster compactness is often linked to grape quality and health status.

The non-invasive nature of the system could also enable an early identification of very compact clusters before harvest in order to establish strategies against fungal diseases, such as botrytis.

It is also remarkable that the absence of features non-extracted directly from image analysis in the model opens the possibility of a direct application of the algorithm in new vineyards and varieties, in contrast to previous works. Cubero et al [21] introduced, in the PLS model, the cluster winegrape variety as a feature, which requires collecting additional clusters of the variety whose compactness is going to be estimated, evaluating its compactness following the OIV method, and re-training the model. Chen et al. [22] introduced features derived from the cluster mass measured by a weighing sensor, which requires prior harvesting of clusters.

### 3.5. Future Work

While the current state of the system is capable of estimating compactness in commercial vineyards under uncontrolled field conditions, some improvements still can be made. The algorithm works properly only for red winegrape varieties due to the initial segmentation step, which uses only color information. In this regard, any white grape pixel could be easily misclassified as leaves or “rachis” pixels. A more robust segmentation algorithm could be developed to overcome this problem, combining color and texture information or recurring deep learning techniques. Also, these solutions could help to develop a fully automated system.

The compactness estimation model would also benefit from a more advanced image analysis algorithm capable of extracting features representing the deformation of berries (to increase the accuracy in detecting highly compact clusters), the degree of occlusion of the cluster (to avoid estimations on highly occluded clusters), and to separate overlapped clusters (to enable an individualized estimation on each cluster of the overlapped set).

## 4. Conclusions

The results of this work show that the developed system was able to estimate winegrape cluster compactness using RGB computer vision on-the-go (at 5 km/h using a mobile sensing platform) and machine learning technology under field conditions. This system enabled a semi-automated, non-invasive method for compactness estimation of a high number of red grapevine clusters under field conditions with low time-consumption. It could be applied to determine the spatial variability of cluster compactness in commercial vineyards, which could be used as new quality input to drive decisions on harvest classification or differential fungicide spraying, for example. The developed methodology constitutes a new tool to improve decision making in precision viticulture, which could be helpful for the wine industry.

## Figures and Tables

**Table 1:** Hyperparameter range considered for each classifier and the used final values.

	Kernel Function (fixed)	Optimized Hyperparameters Range		Final Values	
**SVM**	Radial basis function (RBF)	*Box Constraint*	*Kernel scale*	*Box Constraint*	*Kernel scale*
		[10^−3^, 10^3^]	[10^−3^, 10^3^]	1.4654	24.628
**GPR**	Exponential	*Sigma*	*Kernel scale*	*Sigma*	*Kernel scale*
		[10^−4^, 22.5184]	[0.1216, 121.6122]	0.83194	91.5821

SVM: support vector machine; GPR: Gaussian process regression.

**Table 2:** Performance results of each multinomial logistic regression model for segmenting images in their respective dataset using a 10-fold stratified cross validation on the manually labeled pixel sets in terms of sensitivity, specificity, F1 score, area under the receiver operating characteristic (ROC) curve (AUC) and intersect over union (IoU) metrics. Their average was compared with the performance of a single segmentation with all datasets combined using a 5-fold and a 10-fold stratified cross validation.

Vineyard Canopy Class	T16	G17	Dataset	CS18	T18	Average	5-Fold CV	10-Fold CV
			S18					
	**Sensitivity**							
Trellis	0.9420	0.8760	0.9500	0.9200	0.9760	0.9328	0.5284	0.6700
Gap	0.9740	0.9900	0.9920	0.9960	0.9940	0.9892	0.9868	0.9880
Leaf	0.9760	0.9340	0.9060	0.9680	0.9700	0.9508	0.7476	0.8980
Shoot	0.9440	0.9520	0.9880	1.0000	0.9900	0.9748	0.8992	0.9208
Rachis	0.8640	0.9020	0.9220	0.9660	0.9580	0.9224	0.6912	0.7964
Trunk	0.9020	0.9560	0.9540	0.9660	0.9980	0.9552	0.3620	0.6992
Grape	0.9320	0.9720	0.9700	0.9540	0.9800	0.9616	0.7652	0.8732
	**Specificity**							
Trellis	0.9897	0.9883	0.9930	0.9883	0.9970	0.9913	0.9554	0.9648
Gap	0.9950	0.9987	0.9967	0.9990	0.9977	0.9974	0.9955	0.9963
Leaf	0.9960	0.9893	0.9897	0.9960	0.9947	0.9931	0.9796	0.9829
Shoot	0.9900	0.9963	0.9983	0.9997	0.9987	0.9966	0.9335	0.9789
Rachis	0.9813	0.9850	0.9810	0.9927	0.9947	0.9869	0.9181	0.9673
Trunk	0.9803	0.9820	0.9923	0.9933	0.9987	0.9893	0.9151	0.9423
Grape	0.9900	0.9907	0.9960	0.9927	0.9963	0.9931	0.9661	0.9751
	**F1 Score**							
Trellis	0.9401	0.9003	0.9538	0.9246	0.9789	0.9396	0.5884	0.7123
Gap	0.9721	0.9910	0.9861	0.9950	0.9900	0.9868	0.9801	0.9831
Leaf	0.9760	0.9349	0.9207	0.9719	0.9690	0.9545	0.7996	0.8978
Shoot	0.9421	0.9645	0.9890	0.9990	0.9910	0.9771	0.7826	0.8996
Rachis	0.8745	0.9056	0.9057	0.9612	0.9628	0.9220	0.6334	0.7993
Trunk	0.8931	0.9264	0.9540	0.9631	0.9950	0.9463	0.3869	0.6836
Grape	0.9357	0.9586	0.9729	0.9550	0.9790	0.9602	0.7773	0.8634
	**AUC**							
Trellis	0.9658	0.9322	0.9715	0.9542	0.9865	0.9620	0.7419	0.8174
Gap	0.9845	0.9943	0.9943	0.9975	0.9958	0.9933	0.9912	0.9922
Leaf	0.9860	0.9617	0.9478	0.9820	0.9823	0.9720	0.8636	0.9405
Shoot	0.9670	0.9742	0.9932	0.9998	0.9943	0.9857	0.9164	0.9499
Rachis	0.9227	0.9435	0.9515	0.9793	0.9763	0.9547	0.8047	0.8818
Trunk	0.9412	0.9690	0.9732	0.9797	0.9983	0.9723	0.6386	0.8207
Grape	0.9610	0.9813	0.9830	0.9733	0.9882	0.9774	0.8656	0.9241
	**IoU**							
Trellis	0.8870	0.8187	0.9117	0.8598	0.9587	0.8872	0.4169	0.5532
Gap	0.9456	0.9821	0.9725	0.9901	0.9803	0.9741	0.9610	0.9667
Leaf	0.9531	0.8778	0.8531	0.9453	0.9399	0.9139	0.6661	0.8146
Shoot	0.8906	0.9315	0.9782	0.9980	0.9821	0.9561	0.6428	0.8175
Rachis	0.7770	0.8275	0.8276	0.9253	0.9283	0.8571	0.4635	0.6657
Trunk	0.8068	0.8628	0.9120	0.9288	0.9901	0.9001	0.2399	0.5193
Grape	0.8792	0.9205	0.9473	0.9138	0.9589	0.9239	0.6358	0.7596

**Table 3:** Performance results of the support vector machine classifier for cluster detection validated with the external set and performing a 5-fold cross validation on the whole data for several *k* values of *k*-means tested in terms of sensitivity, specificity, F1 score, and AUC metrics.

		Sensitivity	Specificity	F1 Score	AUC
**Test Set**	*k* = 10	0.760	0.660	0.724	0.751
	*k* = 50	0.738	0.770	0.750	0.828
	*k* = 100	0.765	0.795	0.777	0.865
	*k* = 150	0.750	0.770	0.758	0.841
	*k* = 200	0.720	0.765	0.737	0.848
**5-Fold CV**	*k* = 10	0.811	0.678	0.761	0.821
	*k* = 50	0.804	0.788	0.798	0.884
	*k* = 100	0.821	0.781	0.805	0.903
	*k* = 150	0.790	0.805	0.796	0.888
	*k* = 200	0.799	0.813	0.804	0.902

CV: cross validation.
